# Nutritive value and ruminal degradation of seven Chinese herbs as forage for Tan sheep

**DOI:** 10.1080/21655979.2020.1834740

**Published:** 2020-10-21

**Authors:** Biwei Jiang, Yuxiang Zhou, Tian Wang, Fei Li

**Affiliations:** a Ningxia University, Yinchuan, China; b Ningxia Polytechnic, Yinchuan, China

**Keywords:** Chinese herbs, degradation, nutritive value, Tan sheep

## Abstract

Tan sheep is an indigenous ovine breed of China known for its high meat quality and pleasing taste. Seven herbs of traditional Chinese medicine, namely, *Ephedra sinica, Glycyrrhiza uralensis, Caragana korshinskii, Allium mongolicum, Thymus vulgaris, Astragalus membranaceus*, and *Lespedeza bicolor* are commonly grazed by Tan sheep. It has been widely believed that these herbs are of high nutritive value, which may significantly contribute to the high meat quality and distinct flavor of Tan sheep. However, the nutritive values of these herbs have not been evaluated to date. In this study, samples of the seven herbs were collected from the steppe of Yanchi County of Ningxia Autonomous Region of China. The dry matter (DM), crude protein (CP), ether extract (EE), ash (Ash), calcium (Ca), phosphorus (P), neutral detergent fiber (NDF), and acid detergent fiber (ADF) of these herbs were measured using locally cultivated alfalfa as the standard forage. Digestion of the dry matter, neutral detergent fiber, acid detergent fiber, and crude protein in the rumen of Tan sheep was examined using the nylon bag method, in order to evaluate their feeding nutritional value. Our results show that all the seven herbs meet the nutritional needs of ruminants based on the standard forage alfalfa. However, *Ephedra, Glycyrrhiza, Caragana, Allium, Astragalus*, and *Lespedeza* have higher nutritive value than *Thymus* (*P* < 0.05). According to the ruminal degradation rates of dry matter, neutral detergent fiber, acid detergent fiber, and crude protein, the nutritive value of *Caragana, Allium*, and *Lespedeza* is higher than that of *Ephedra, Glycyrrhiza, Astragalus*, and *Thymus* (*P* < 0.05). The overall nutritive value of *Allium* is the highest among the seven herbs and therefore *Allium* is recommended to better meet the nutritional needs of Tan sheep.

## GRAPHICAL ABSTRACT

**Figure:**
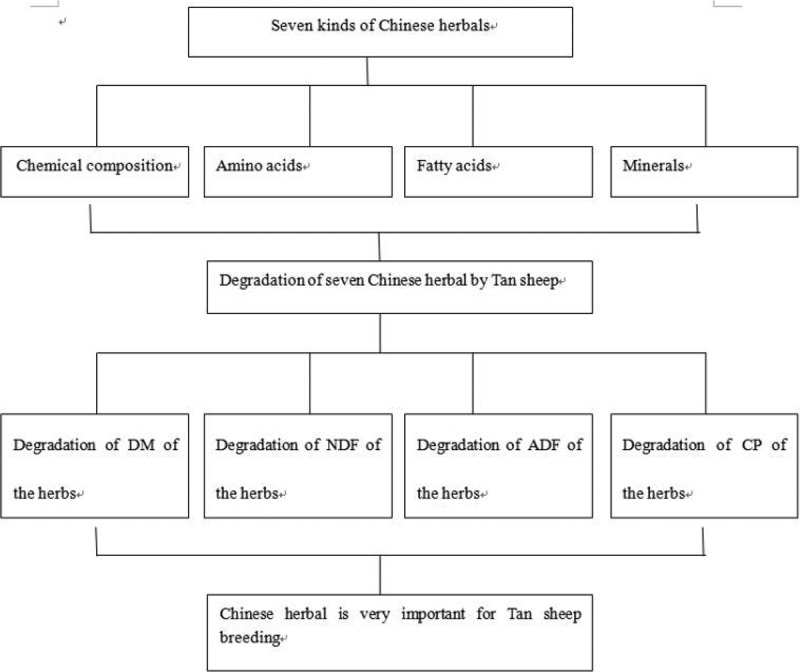

## 1. Introduction

Tan sheep is an indigenous ovine breed of Ningxia Hui Autonomous Region of China known for its high meat quality and taste [1]. Yanchi County of Ningxia Autonomous Region is the designated national conservation region for Tan sheep and has a total of 476,100 hm^2^ of natural pastures characterized by a variety of indigenous plant species. Yanchi County is located in the arid/semiarid steppe grasslands of northern China, and thus the Tan sheep industry is highly dependent on these indigenous plant species that have adapted to the local climate [2]. Among these indigenous plant species are seven herbs used in traditional Chinese medicine, namely, *Ephedra sinica, Glycyrrhiza uralensis, Caragana korshinskii, Allium mongolicum, Thymus vulgaris, Astragalus membranaceus*, and *Lespedeza bicolor*, which are commonly grazed by Tan sheep. It has been widely believed that the seven herbs are of high nutritive value, which may largely contribute to the high meat quality and distinct flavor of Tan sheep.

As a new type of green feed additive, Chinese herbal medicine feed additive has many advantages, such as promoting animal growth, improving product quality, improving body disease resistance, and so on. It is safe and nontoxic. It is widely used in animal breeding production. Chinese herbal feed additives can improve feed intake and feed digestibility of pregnant ewes, protect pregnancy and prevent abortion, increase lambing rate, survival rate of lambs and weight of newborn lambs, and improve immunity and anti-stress ability of pregnant ewes [3]. The objective of this paper is to evaluate the nutritive value of these herbs. Evaluation of the nutritive values of forage plants is of great significance to the rational use of pasture resources and ecosystem management [4]. The quality of forages is affected by the digestibility of plant materials in grazing livestock [5]. Indigestible components such as fibrous compounds reduce not only the amount of digestible materials but also the palatability of forages [6]. Therefore, multiple nutritive metrics should be considered in combination with digestibility metrics to evaluate the quality of forages [7]. Here, we employed multiple nutritive metrics to evaluate the nutritive value of the seven herbs. Degradation of nutrients in the rumen of Tan sheep was also examined using the nylon bag technique [8]. The related research has not been reported. The research on the feeding value of Chinese herbal medicine will be of great significance to Tan sheep breeding.

## 2. Materials and methods

All experimental procedures involving animal care and use were conducted according to the Regulations for the Administration of Affairs Concerning Experimental Animals (The State Science and Technology Commission of P.R. China, 1988). This study was approved by the Animal Care and Use Committee of Ningxia University (SYXK (Ning) 2019–0522).

### 2.1. Sample collection

Samples of the seven Chinese herbs, including *E. sinica, G. uralensis, C. korshinskii, A. mongolicum, T. vulgaris, A. membranaceus*, and *L. bicolor*, were collected from the steppes of Dashuikeng, Yanchi County of Ningxia, China on July 12–13, 2019 when the rain is abundant, the temperature is warm, and most of the pastures are in the period between rapid growth and early flowering. These herbs are healthy and wild. Locally cultivated alfalfa was collected as control. Only the tender stems and leaves were collected to simulate sheep feeding, and samples were then dried in the laboratory.

### 2.2. Chemical analysis

Chemical analysis reagents were purchased from Yinchuan, Ningxia, China. Dry matter (DM), Crude protein (CP), Ether extract (EE), Crude ash (Ash), Ca, P, Neutral detergent fiber (NDF), and Acid detergent fiber (ADF) were determined according to the Chinese Standard GB/T 6435–1986 [9] and Van Soest [10]. Amino acid (AA) type and content were determined using an L-8800 amino acid automatic analyzer according to the Chinese Standard GB/T 18246–2000 [11]. Fatty acids were measured using hydrolysis extraction-gas chromatography according to Chinese Standard GB/T22223-2008 [12]. Mineral elements such as copper, iron, and magnesium were measured using an AA370MC flame atomic absorption spectrophotometer (FAAS) according to the Chinese Standard GB/T18932.12–2002 [13]. Selenium was determined using AA370MC FAAS according to Chinese Standard GB/T13883-2008 [14], and chromium was determined by AA370MC FAAS according to the Chinese Standard GB13078-2001 [15].

### 2.3. Animals and feeding

Three healthy castrated Ningxia Tan sheep (1–1.5 years old, weighing 30 kg) were selected. The sheep were fed three times a day and provided with ad libitum access to drinking water for 50 days to enhance the body condition before the start of the experiment. Sheep diet is formulated according to Table 1, and the feeding standard was based on the needs for a daily weight gain of 120 g in a 24–31 kg adult weather.

**Table 1.** Formulation of diet

Ingredients	Dry matter (kg/d)	Feeding level	
Silage	0.3300	Metabolic energy (MJ/kg)	8.4000
Hay	0.3000	crude protein (g/d)	130.0000
Corn	0.1800	Calcium (g/d)	4.5000
Soybean meal	0.0889	Phosphorus (g/d)	3.0000
Bran	0.0170		
Sunflower cake	0.0100		
Premix	0.0300		
Salt	0.0020		
Total	0.9579		
Feeding standard	0.9000		

### 2.4. In situ rumen incubation of feeds

The forage samples were pulverized through a 40-mesh standard sieve in the laboratory using a pulverizer. Approximately 2 g of the sample was placed in a nylon bag. Two nylon bags were clamped on a long 18-cm semi-soft plastic tube and tied with a rubber band. Before the morning feeding (around 8:00), the bag was sent to the ventral sac of the rumen through a ruminal cannula. To prevent the nylon bag from falling off, the other end of the plastic tube was tied with a nylon cord on the iron ring of the rumen fistula cap. Seven tubes were placed in the rumen of each sheep for a total of 14 bags. One tube was taken out at time points of 3, 6, 12, 24, 36, 48, and 72 h. The removed nylon bag, along with the plastic tube, was rinsed with tap water for about 5 min until the water was clear. The washed nylon bag was dried in a 65°C oven to a constant weight (approximately 48 h). Samples were stored in the laboratory and analyzed in terms of DM, CP, NDF, and ADF content. A control bag was soaked in water at 39°C for 1 h, rinsed, dried, and used to correct the loss of materials during washing.

### 2.5. Calculations and statistical analyses

The rumen degradation rate of a nutrient (e.g. DM, CP, NDF, and ADF) at a certain time point is calculated using the equation: P = (a − b)/a × 100, where P is the degradation rate (%) of nutrients (DM, NDF, ADF, CP) in the rumen at a certain time point; a is the total amount of a nutrient in the nylon bag before placed into the rumen (DM, NDF, ADF, and CP); and b is the total amount of a nutrient (DM, NDF, ADF, and CP) remaining in the nylon bag after rumen incubation.

The rumen dynamic degradation rate was calculated by nonlinear procedures of SAS statistical software using the equation: dp = a + b (1 − e^−ct^), where dp is the degradation rate (%) of DM, CP, NDF, and ADF after time t; a is the ratio of rapidly degrading components in the sample, which is the intercept of the exponential curve in the equation; b is the ratio of sample; c is the degradation constant (%/h) of the slowly degrading components; t is the incubation time (h) of the sample in the rumen; and the a, b, and c constants are calculated by the least squares method. The effective degradation rate of nutrients in the feeds was calculated using the equation: p = a + b × c/(c + k), where p is the dynamic degradation rate of DM, CP, NDF, and ADF in the feeds, and k is the speed of chyme outflow. The data were first processed using Excel. ANOVA was conducted using SAS (8.2) software (SAS Institute, Cary, NC). The multiple comparison of the mean values was performed using the Duncan method.

## 3. Results and discussion

### 3.1. Chemical composition

The chemical composition of alfalfa and the seven herbs is shown in Table 2. The content of DM in seven Chinese herbals ranged from 40.99 to 41.36; EE ranged from 2.29 to 6.19; CP ranged from 9.00 to 33.31; Ash ranged from 6.92 to 23.40; Ca ranged from 0.84 to 2.16; P ranged from 0.16 to 0.44; NDF ranged from 19.15 to 43.84; ADF ranged from 18.40 to 39.10. Forages of high level of crude proteins and low levels of NDF and ADF are considered to be of high nutritive value. Our results indicate that all the seven herbs should be able to meet the nutritional needs of ruminants based on the data of the standard forage alfalfa. However, *E. sinica, G. uralensis, C. korshinskii, A. mongolicum, A. membranaceus*, and *L. bicolor* have higher nutritive value than *T. vulgaris*.

**Table 2.** Chemical composition of seven Chinese herbs (%)

Species^1^	DM^3^	EE/DM^3^	CP/DM^3^	ASH/D^2^M	Ca/DM^3^	P/DM^3^	NDF/D^2^M	ADF/D^2^M
*E.s.*	41.36 ± 0.40 ^Aa^	2.29 ± 0.05 ^G^	14.14 ± 0.5 0 ^ab^	8.03 ± 0.11 ^Fa^	1.74 ± 0.02 ^B^	0.16 ± 0.00 ^Fd^	39.12 ± 0.03 ^a^	36.20 ± 0.20 ^B^
*G.u.*	33.88 ± 0.57 ^Bb^	6.19 ± 0.01 ^A^	18.81 ± 0.08 ^D^	23.40 ± 0.33 ^A^	1.26 ± 0.03 ^b^	0.23 ± 0.00 ^Ca^	34.10 ± 0.87 ^D^	31.71 ± 0.30 ^Cb^
*C.k.*	30.79 ± 0.51 ^C^	2.49 ± 0.04 ^F^	21.93 ± 0.29 ^C^	6.92 ± 0.01 ^G^	0.86 ± 0.08 ^F^	0.21 ± 0.01 ^CDb^	32.94 ± 0.01 ^E^	32.03 ± 0.23 ^ab^
*A.m.*	7.45 ± 0.28 ^E^	5.50 ± 0.02 ^C^	33.31 ± 0.09 ^B^	15.95 ± 0.21 ^B^	1.00 ± 0.01 ^E^	0.44 ± 0.01 ^A^	19.15 ± 0.04 ^Fb^	18.40 ± 0.08 ^D^
*T.v.*	40.89 ± 0.22 ^a^	5.90 ± 0.03 ^B^	9.00 ± 0.03 ^F^	12.10 ± 0.18 ^C^	1.50 ± 0.01 ^Ca^	0.18 ± 0.03 ^EFc^	43.84 ± 0.04 ^A^	39.10 ± 0.08 ^A^
*A.me.*	33.33 ± 0.26 ^b^	3.45 ± 0.04 ^Da^	13.48 ± 0.17 ^Eb^	8.25 ± 0.03 ^Fa^	1.30 ± 0.00 ^Db^	0.19 ± 0.02 ^DEc^	39.52 ± 0.04 ^Ba^	32.53 ± 0.43 ^Ca^
*L.b.*	40.99 ± 0.93 ^a^	3.38 ± 0.02 ^a^	14.33 ± 0.08 ^Ea^	8.75 ± 0.01 ^E^	2.16 ± 0.01 ^A^	0.16 ± 0.01 ^EFd^	35.70 ± 0.04 ^C^	31.42 ± 0.56 ^b^
Alfalfa	20.38 ± 0.10 ^D^	2.77 ± 0.04 ^E^	35.24 ± 0.19 ^A^	11.24 ± 0.09 ^D^	1.47 ± 0.02 ^a^	0.39 ± 0.07 ^B^	18.65 ± 0.39 ^b^	14.64 ± 0.35 ^E^

^1^*E.s. = Ephedra sinica, G.u. = Glycyrrhiza uralensis, C.k. = Caragana korshinskii, A.m. = Allium mongolicum, T.v. = Thymus vulgaris, A.me. = Astragalus membranaceus, L.b. = Lespedeza bicolor*, DM = dry matter, EE = ether extract, CP = crude protein, ASH = ash, NDF = neutral detergent fiber, ADF = acid detergent fiber.

^2^The same superscript lowercase letters indicate *P* > 0.05; the different superscript lowercase letters indicate 0.01 < *P* < 0.05; the different superscript uppercase letters indicate *P* < 0.01.

^3^DM is based on fresh weight.

### 3.2. Amino acids

The amino acid profiles of the seven herbs are shown in Table 3. The composition and proportion of the amino acids are key to the accurate evaluation of protein quality of feedstuffs. An imbalance in amino acids, especially the essential amino acids, prevents responses to increased dietary crude proteins and affects nitrogen utilization of feedstuffs [16]. The Food and Agriculture Organization of the United Nations (FAO) and the World Health Organization (WHO) [17] recommend about 40% Essential amino acids (EAA)/Total amino acid (TAA) for better quality protein. Our results indicate that the seven herbs are of high protein quality as judged by amino acid content and essential amino acids. The protein quality of *A. mongolicum* was the highest, while that of *T. vulgaris* was the lowest.

**Table 3.** Amino acid (AA) contents of seven Chinese herbs (AA/DM ×%)

	*E.s.*	*G.u.*	*C.k.*	*A.m.*	*T.v.*	*A.me.*	*L.b.*	Alfalfa
Asp	1.23 ± 0.06^DEa^	1.00 ± 0.01 ^Eb^	2.22 ± 0.00 ^C^	3.28 ± 0.22^B^	0.83 ± 0.02^Fb^	1.36 ± 0.03^a^	1.41 ± 0.08 ^Da^	4.47 ± 0.06^A^
Thr	0.57 ± 0.02 ^c^	0.62 ± 0.01^bc^	0.86 ± 0.00^B^	1.32 ± 0.08^a^	0.43 ± 0.01^D^	0.78 ± 0.00^B^	0.65 ± 0.03 ^Cb^	1.34 ± 0.01^Aa^
Ser	0.67 ± 0.06^de^	0.68 ± 0.02^de^	0.99 ± 0.01^Bb^	1.40 ± 0.13^Aa^	0.51 ± 0.00^Ee^	0.87 ± 0.01^BCc^	0.74 ± 0.02 ^CDd^	1.24 ± 0.01^Ab^
Glu	1.66 ± 0.11^ab^	1.49 ± 0.06 ^CDbc^	1.88 ± 0.05^Ca^	5.59 ± 0.35^A^	1.17 ± 0.04^Dc^	1.76 ± 0.00^Cab^	1.46 ± 0.05^bc^	2.77 ± 0.04^B^
Gly	0.50 ± 0.01^DEc^	0.48 ± 0.01 ^cd^	0.68 ± 0.01^a^	1.00 ± 0.06^B^	0.43 ± 0.01^Ed^	0.72 ± 0.01 ^Ca^	0.59 ± 0.04 ^Db^	1.38 ± 0.01^A^
Ala	0.72 ± 0.03 ^c^	0.75 ± 0.01 ^c^	0.85 ± 0.01^BCb^	1.88 ± 0.11^a^	0.52 ± 0.01^D^	0.92 ± 0.01^Bb^	0.73 ± 0.0 ^c^	1.95 ± 0.04^Aa^
Val	0.84 ± 0.09^bc^	0.63 ± 0.04 ^c^	0.99 ± 0.04^B^	1.72 ± 0.25^Aa^	0.66 ± 0.03^BCc^	0.81 ± 0.00^bc^	0.79 ± 0.01^bc^	1.53 ± 0.02^a^
Met	0.36 ± 0.05^b^	0.24 ± 0.0 ^b^	0.29 ± 0.08^b^	1.31 ± 1.02^a^	0.30 ± 0.02^b^	0.25 ± 0.01^b^	0.24 ± 0.01^b^	0.31 ± 0.00^b^
Ile	0.32 ± 0.03 ^cd^	0.31 ± 0.00 ^cd^	0.47 ± 0.03^bc^	0.82 ± 0.20^a^	0.25 ± 0.01^d^	0.51 ± 0.01^Bb^	0.39 ± 0.02^bcd^	0.92 ± 0.01^Aa^
Leu	0.51 ± 0.03 ^d^	0.69 ± 0.04 ^cd^	0.88 ± 0.14 ^cd^	1.44 ±.50 ^Ba^	0.43 ± 0.03 ^Dd^	1.13 ± 0.01 ^BCbc^	0.80 ± 0.06 ^cd^	1.86 ± 0.02 ^ABab^
Tyr	0.47 ± 0.02^ab^	0.39 ± 0.01^b^	0.54 ± 0.07^ab^	1.40 ± 1.12^a^	0.35 ± 0.01^b^	0.42 ± 0.01^ab^	0.42 ± 0.00^ab^	0.86 ± 0.02^ab^
Phe	0.48 ± 0.10 ^Ccd^	0.55 ± 0.01^BCcd^	0.76 ± 0.02^BCab^	0.94 ± 0.31 ^Ba^	0.41 ± 0.00 ^Cd^	0.73 ± 0.01^BCab^	0.62 ± 0.04^BCcd^	1.37 ± 0.01^A^
Lys	0.71 ± 0.03^d^	0.73 ± 0.02^Ed^	1.05 ± 0.02 ^Ca^	1.50 ± 0.07^B^	0.50 ± 0.01 ^F^	0.95 ± 0.00^b^	0.83 ± 0.03^Dc^	1.73 ± 0.02^A^
His	0.40 ± 0.01 ^cd^	0.28 ± 0.00^Cef^	0.46 ± 0.04^Bc^	0.61 ± 0.11^b^	0.26 ± 0.01 ^Cf^	0.32 ± 0.00^Bdef^	0.37 ± 0.01 ^Ccde^	0.70 ± 0.01^Aa^
Arg	0.40 ± 0.02^Eb^	0.29 ± 0.01 ^F^	0.85 ± 0.03^C^	1.05 ± 0.01^B^	0.37 ± 0.01^b^	0.60 ± 0.01 ^Da^	0.58 ± 0.05^a^	1.53 ± 0.02^A^
Pro	0.77 ± 0.06^CDc^	0.50 ± 0.05^de^	1.09 ± 0.18^BCb^	1.42 ± 0.15 ^Ba^	0.34 ± 0.04^Ee^	0.69 ± 0.01^Dcd^	1.09 ± 0.09^BCb^	2.03 ± 0.03^A^
Trp	0.04 ± 0.04 ^Ba^	0.03 ± 0.01^a^	0.03 ± 0.01^a^	0.02 ± 0.01^a^	0.02 ± 0.01^a^	0.02 ± 0.01^a^	0.02 ± 0.01^a^	0.32 ± 0.07^A^
Total AA (TAA)	10.56 ± 0.61^DEd^	9.58 ± 0.16^Ee^	14.85 ± 0.25^B^	26.63 ± 0.31^Aa^	7.72 ± 0.16 ^F^	12.78 ± 0.08 ^Cb^	11.66 ± 0.50^CDc^	25.94 ± 0.35^a^
CP	14.14 ± 0.50^ab^	18.81 ± 0.08^D^	21.93 ± 0.29 ^C^	33.31 ± 0.09^B^	9.00 ± 0.03 ^F^	13.48 ± 0.17^Eb^	14.33 ± 0.08^Ea^	35.24 ± 0.19^A^
TAA/CP	74.69 ± 4.28^CDEc^	50.92 ± 0.85 ^F^	67.70 ± 1.12^Ed^	79.95 ± 0.95^BDb^	85.79 ± 1.78 ^Ba^	94.79 ± 0.57^A^	81.42 ± 3.50^Cab^	73.61 ± 1.00^DEc^
EAA (Essential AA)	4.24 ± 0.37 ^cd^	4.14 ± 0.02 ^Cd^	5.83 ± 0.01^Bb^	10.43 ± 0.49^Aa^	3.31 ± 0.01^D^	5.55 ± 0.01^b^	4.73 ± 0.16^Cc^	9.90 ± 0.13^a^
EAA/TAA	40.05 ± 1.24^b^	43.15 ± 0.48^a^	39.28 ± 0.55^b^	39.20 ± 2.31^Bb^	42.90 ± 0.70^a^	43.42 ± 0.32^Aa^	40.52 ± 0.33^b^	38.14 ± 0.03^b^
LAA (Limiting AA)	0.86 ± 0.08^bc^	0.92 ± 0.02^bc^	1.17 ± 0.06^bc^	2.75 ± 1.51^Aa^	0.74 ± 0.01^Bc^	1.37 ± 0.00^bc^	1.04 ± 0.04^bc^	2.17 ± 0.02^ABab^
LAA/TAA	8.14 ± 0.31^a^	9.57 ± 0.08^a^	7.87 ± 0.27^a^	10.35 ± 0.80^a^	9.46 ± 0.10^a^	10.72 ± 0.06^a^	8.92 ± 0.00^a^	8.35 ± 0.02^a^
Flavor AA	5.94 ± 0.50^DEb^	5.16 ± 0.06^EFc^	8.05 ± 0.08 ^C^	15.65 ± 0.47^A^	4.37 ± 0.03^Fd^	6.59 ± 0.03 ^Da^	6.00 ± 0.21^DEab^	13.29 ± 0.18^B^
Flavor AA/TAA	56.18 ± 1.52^ab^	53.89 ± 0.34^BCbc^	54.22 ± 1.45^bc^	58.77 ± 2.50^Aa^	56.63 ± 0.78^ABab^	51.56 ± 0.07^Cc^	51.40 ± 0.48 ^c^	51.23 ± 0.01 ^c^

*E.s. = Ephedra sinica, G.u. = Glycyrrhiza uralensis, C.k. = Caragana korshinskii, A.m. = Allium mongolicum, T.v. = Thymus vulgaris, A.me. = Astragalus membranaceus, L.b. = Lespedeza bicolor*, CP = crude protein, AA = amino acid. The same superscript lowercase letters indicate *P* > 0.05; the different superscript lowercase letters indicate 0.01 < *P* < 0.05; the different superscript uppercase letters indicate *P* < 0.01.

### 3.3. Fatty acids

The levels of 14 types of fatty acids in the seven herbs are shown in Table 4. It can be seen from Table 4 that the contents of palmitic acid (c16:0), linoleic acid (c18:2n6c) and α-linolenic acid (c18:3n3) are higher in each sample, among which linoleic acid (c18:2n6c) and α-linolenic acid (c18:3n3) are essential fatty acids. The content of palmitic acid (c16:0) was higher in *Glycyrrhiza uralensis*, *Caragana korshinskii*, *Allium mongolicum,* and *Lespedeza bicolor* (0.50%, 0.52%, 0.42% and 0.48%, respectively), and the difference was not significant (P > 0.05). The content of stearic acid in thyme was the lowest, only 0.05%, which was significantly lower than that of licorice and *Caraganakorshinskii* (0.01 < *P*< 0.05). The content of oleic acid in thyme and *Ephedra* was higher (0.20% and 0.15%, respectively). The content of oleic acid in alfalfa and other flavor plants was less, and the difference was very significant (*P* < 0.01). Essential fatty acids (EFA) included linoleic acid and linolenic acid. Except alfalfa, the content of linoleic acid (c18:2n6c) in *Caragana* was the highest (0.33%), and the lowest was Astragalus membranaceus (0.12%). The content of α – linolenic acid (c18:3n3) in licorice, *Caragana korshinskii*, thyme, and *Lespedeza* was 0.70%, 0.60%, 0.62%, and 0.51%, respectively, which was significantly lower than that of Alfalfa (*P* < 0.01). The content of α – linolenic acid in Allium mongolicum Regel was the lowest, only 0.25%. Fatty acids in the diet can be directly absorbed by the digestive tract of livestock [18]. Linoleic acid is an essential fatty acid (EFA) that cannot be synthesized in animals. Linoleic acid can produce γ-linolenic acid through the eicosapentaenoic acid (EPA, C20:5) pathway and finally produces prostaglandins, which participate in the regulation of a variety of physiological processes, including blood pressure regulation, cholesterol synthesis, and cell proliferation [19]. Studies have shown that the α-linolenic acid content in pig adipose tissues is highly correlated with the α-linolenic acid content in the diet, and the fatty acid composition of pig adipose tissues can be predicted based on the fatty acid composition of the diet [20]. Palmitic acid is a saturated fatty acid, and appropriate consumption is beneficial to fat metabolism; however, excessive consumption of palmitic acid is the main cause of fat deposition in animals. Our results revealed that the content of *trans* fatty acids (TFA) is the highest in *G. uralensis* and *C. korshinskii*, whereas the EFA contents were similar among all seven herbs. The content of stearic acid, which is related to the unpleasant flavor of sheep meat, is low, and the contents of palmitic acid, oleic acid, linoleic acid, and linolenic acid are high. Therefore, seven herbs as forage may affect the fatty acid content of Tan sheep, which might contribute to the meat quality.

**Table 4.** Fatty acid (FA) contents of seven Chinese herbs (FA/DM ×%)

	*E.s.*	*G.u.*	*C.k.*	*A.m.*	*T.v.*	*A.me.*	*L.b.*	Alfalfa
Lauric acid (C12:0)	0.02 ± 0.01^a^	0.00^b^	0.01 ± 0.00^ab^	0.01 ± 0.00^ab^	0.01 ± 0.00^ab^	0.00^b^	0.01 ± 0.01^ab^	0.00^b^
Myristic acid (C14:0)	0.02 ± 0.01^a^	0.07 ± 0.08^a^	0.02 ± 0.01^a^	0.05 ± 0.03^a^	0.01 ± 0.00^a^	0.03 ± 0.01^a^	0.02 ± 0.01^a^	0.00^a^
Palmitic acid (C16:0)	0.37 ± 0.02^Bc^	0.50 ± 0.01^ab^	0.52 ± 0.01^a^	0.52 ± 0.04^Aa^	0.33 ± 0.02^Bcd^	0.31 ± 0.02^Bd^	0.48 ± 0.03^ab^	0.45 ± 0.03^Ab^
Palmitoleic acid (C16:1n7)	0. 00^Ca^	0.01 ± 0.00^B^	0. 00^a^	0. 00^a^	0. 00^a^	0. 00^a^	0. 00^a^	0.03 ± 0.01^A^
Stearic acid (C18:0)	0.09 ± 0.04^ab^	0.11 ± 0.01^a^	0.10 ± 0.00^a^	0.08 ± 0.01^ab^	0.05 ± 0.01^b^	0.10 ± 0.01^a^	0.09 ± 0.01^ab^	0.08 ± 0.00^ab^
Oleic acid (C18:1n9c)	0.15 ± 0.01^B^	0.08 ± 0.01^Ca^	0.07 ± 0.01^a^	0.05 ± 0.03^a^	0.20 ± 0.02^A^	0.07 ± 0.00^a^	0.07 ± 0.00^a^	0.05 ± 0.01^a^
Linoleic acid (C18:2n6c)	0.27 ± 0.02^b^	0.28 ± 0.01^b^	0.33 ± 0.03^ABab^	0.28 ± 0.03^b^	0.30 ± 0.02^b^	0.12 ± 0.01^C^	0.30 ± 0.01^b^	0.39 ± 0.01^Aa^
α-linolenic acid (C18:3n3)	0.33 ± 0.02^EFc^	0.70 ± 0.01 ^Ba^	0.60 ± 0.04^b^	0.25 ± 0.01 ^Fd^	0.62 ± 0.03^BCb^	0.38 ± 0.01^Ec^	0.51 ± 0.01^D^	1.21 ± 0.05^A^
Arachidonic acid (C20:0)	0.14 ± 0.02^A^	0.04 ± 0.00^bc^	0.07 ± 0.02 ^Ba^	0.05 ± 0.00^BCab^	0.04 ± 0.00^BCbc^	0.03 ± 0.01^bc^	0.02 ± 0.00^Cc^	0.03 ± 0.01 ^c^
Heneicosanoic acid (C21:0)	0.12 ± 0.02^C^	0.28 ± 0.03^A^	0.00 ^Da^	0.00^a^	0.00^a^	0.22 ± 0.00^B^	0.00^a^	0.00^a^
Cis-11-icoic acid (C20:1)	0.01 ± 0.01^a^	0.00^a^	0.00^a^	0.00^a^	0.00^a^	0.00^a^	0.00 ^a^	0.00^a^
Behenic acid (C22:0)	0.01 ± 0.00 ^Cd^	0.18 ± 0.04^Aa^	0.11 ± 0.01^ABb^	0.06 ± 0.04 ^cd^	0.08 ± 0.01^BCbc^	0.03 ± 0.01 ^cd^	0.06 ± 0.02^BCcd^	0.03 ± 0.02^Ccd^
Cis-13,16-docosadienoic acid (C22:2n6)	0.01 ± 0.00 ^c^	0.01 ± 0.00 ^c^	0.00 ^c^	0.00 ^c^	0.00 ^c^	0.06 ± 0.01^Aa^	0.01 ± 0.01 ^Bc^	0.05 ± 0.01^Ab^
Lignoceric acid (C24:0)	0.12 ± 0.01^Aa^	0.11 ± 0.01^a^	0.10 ± 0.01^a^	0.04 ± 0.01^Bb^	0.03 ± 0.01^b^	0.04 ± 0.00^b^	0.04 ± 0.00^b^	0.03 ± 0.01^b^
Total fatty acid (TFA)	1.62 ± 0.18^bc^	2.36 ± 0.19^Aa^	1.93 ± 0.13^ABb^	1.37 ± 0.20 ^c^	1.64 ± 0.13^BCbc^	1.38 ± 0.11 ^Cc^	1.59 ± 0.08^bc^	2.36 ± 0.10 ^a^
Saturated fatty acid (SFA)	0.86 ± 0.10^BCa^	1.29 ± 0.16^A^	0.93 ± 0.07 ^Ba^	0.80 ± 0.13^BCab^	0.53 ± 0.06^Cc^	0.76 ± 0.06^BCabc^	0.70 ± 0.06^abc^	0.60 ± 0.03^BCbc^
SFA/TFA	53.35 ± 0.17^Bb^	54.44 ± 2.47^b^	48.36 ± 0.49 ^C^	58.29 ± 0.96^Aa^	32.29 ± 0.81^E^	54.71 ± 0.32^ABb^	44.14 ± 1.32^D^	25.42 ± 0.08 ^F^
Unsaturated fatty acid (USFA)	0.76 ± 0.08^DEde^	1.07 ± 0.03^ABb^	0.99 ± 0.06^ABbc^	0.57 ± 0.07^Ef^	1.11 ± 0.07^Bb^	0.62 ± 0.04^Eef^	0.89 ± 0.02^CDcd^	1.76 ± 0.07^Aa^
USFA/TFA	46.65 ± 0.17^Ea^	45.56 ± 2.47^EFa^	51.65 ± 0.49^D^	41.71 ± 0.96^Fb^	67.71 ± 0.81^B^	45.30 ± 0.31^a^	55.86 ± 1.32 ^C^	74.58 ± 0.09^A^
Essential fatty acid (EFA)	0.60 ± 0.08^Cc^	0.98 ± 0.02 ^Ba^	0.93 ± 0.05^Bab^	0.52 ± 0.04 ^c^	0.92 ± 0.06^ab^	0.50 ± 0.03 ^c^	0.81 ± 0.01^Bb^	1.60 ± 0.06^A^
EFA/T)	36.89 ± 0.61^DEc^	41.57 ± 2.60 ^Db^	48.09 ± 0.33^Ca^	38.33 ± 2.41^DEbc^	55.98 ± 1.08^B^	36.30 ± 0.67^Ec^	50.90 ± 1.57^a^	67.64 ± 0.06^A^

*E.s*. =* Ephedra sinica, G.u*. = *Glycyrrhiza uralensis, C.k. *=* Caragana korshinskii, A.m. *=* Allium mongolicum, T.v*. =* Thymus vulgaris, A.me*. =* Astragalus membranaceus, L.b. *= *Lespedeza bicolor*. The same superscript lowercase letters indicate *P* > 0.05; the different superscript lowercase letters indicate 0.01 < *P* < 0.05; the different superscript uppercase letters indicate *P* < 0.01.

### 3.4. Minerals

The contents of various mineral elements in the seven herbs are shown in Table 5. The mineral elements such as Cu, Fe, Mg, Cr, Se are essential trace elements in animals, which play an important role in the normal physiological and biochemical process of animals and achieve the purpose of improving meat quality in their own unique way. The minerals and trace elements obtained from forages are crucial to the health of the livestock. The contents of five minerals (copper, iron, magnesium, chromium, and selenium) were higher in *T. vulgaris, G. uralensis, A. mongolicum*, and *L. bicolor* than in the other three herbs and alfalfa. Except for *G. uralensis*, the content of magnesium in the other herbs and alfalfa reached >3,000 mg/kg. The contents of copper, iron, and chromium in *A. membranaceus* were the lowest among the seven herbs.

**Table 5.** Mineral contents of seven Chinese herbs (minerals/DM, mg/kg)

	Cu	Fe	Mg	Gr	Se
*E.s.*	15.45 ± 0.11^E^	244.36 ± 0.62 ^G^	3832 ± 4.48 ^C^	1.141 ± 0.02^a^	2.212 ± 0.02^Ec^
*G.u.*	20.88 ± 0.01^B^	2735.20 ± 2.00^A^	1863 ± 1.51 ^F^	0.647 ± 0.02^C^	7.642 ± 0.01^A^
*C.k.*	18.00 ± 0.25^D^	682.38 ± 0.03 ^D^	3393 ± 4.46^a^	0.363 ± 0.01^Ed^	2.527 ± 0.13^CDa^
*A.m.*	18.59 ± 0.06^C^	363.07 ± 1.46^E^	3482 ± 3.08^E^	1.150 ± 0.02 ^Ba^	2.375 ± 0.02 ^Db^
*T.v.*	40.48 ± 0.12^A^	2350.03 ± 0.11^B^	3969 ± 3.06^B^	0.579 ± 0.01 ^Db^	2.069 ± 0.01^Ed^
*A.me.*	2.72 ± 0.01 ^H^	227.17 ± 0.06 ^H^	3483 ± 9.14 ^Da^	0.321 ± 0.01^Ee^	3.792 ± 0.02^B^
*L.b.*	12.20 ± 0.05 ^F^	1227.65 ± 0.01^C^	3996 ± 1.50^A^	0.536 ± 0.01^Dc^	2.560 ± 0.03^Ca^
Alfalfa	10.16 ± 0.05 ^G^	324.65 ± 0.11 ^F^	3442 ± 6.05^a^	1.801 ± 0.02^A^	1.235 ± 0.01^F^

*E.s*. =* Ephedra sinica, G.u*. = *Glycyrrhiza uralensis, C.k. *=* Caragana korshinskii, A.m. *=* Allium mongolicum, T.v*. =* Thymus vulgaris, A.me*. =* Astragalus membranaceus, L.b. *= *Lespedeza bicolor*, DM = dry matter. The same superscript lowercase letters indicate *P* > 0.05; the different superscript lowercase letters indicate 0.01 < *P* < 0.05; the different superscript uppercase letters indicate *P* < 0.01.

### 3.5. Degradation of DM of the herbs

**Figure 1.**
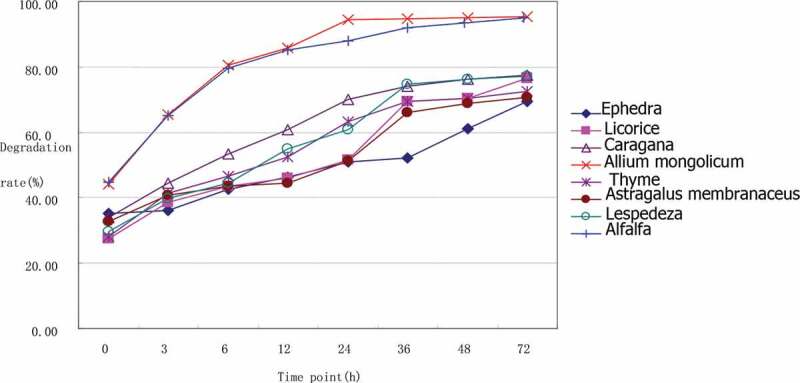
Ruminal dry matter degradation of seven Chinese herbs

Table 6 shows our 72-h time course analysis of rumen degradation found that the ruminal degradation rate of DM of the seven herbs and alfalfa ranked from high to low was as follows: *A. mongolicum* > alfalfa > *C. korshinskii* > *L. bicolor* > *T. vulgaris* > *G. uralensis* > *A. membranaceus* > *E. sinica* (Table 6 and Figure 1). It can be seen from Figure 1 that the degradation curves of *Allium mongolicum*, *Medicago sativa*, *Caraganakorshinskii*, and *Lespedeza bicolor* almost coincide in 36–72 h. The degradation rates of Allium mongolicum and alfalfa were significantly higher than those of other flavor plants (*P* < 0.01). After 72 hours of degradation, the dry matter degradation rate of Allium mongolicum and alfalfa reached more than 95%, and the difference was not significant (*P* > 0.05), but it was significantly higher than other flavor plants (*P* < 0.01).

**Table 6.** Ruminal dry matter degradation of seven Chinese herbs (%)

DM	*E.s.*	*G.u.*	*C.k.*	*A.m.*	*T.v.*	*A.me.*	*L.b.*	Alfalfa
a^1^	36.49 ± 0.21^Bb^	31.26 ± 0.11^d^	34.74 ± 0.47 ^c^	44.49 ± 1.84^a^	30.56 ± 0.55^d^	34.67 ± 1.15^Bc^	31.23 ± 0.31 ^Cd^	44.93 ± 1.10^Aa^
b^1^	60.18 ± 1.24^A^	51.96 ± 1.08 ^Ba^	41.89 ± 0.25^EFd^	50.25 ± 1.94^BCa^	41.51 ± 0.73^Fd^	44.09 ± 0.97^Ec^	47.91 ± 0.30^CDb^	47.19 ± 0.68 ^Db^
c^1^	0.01 ± 0.00^Ef^	0.03 ± 0.00^De^	0.09 ± 0.01^Bb^	0.18 ± 0.01^Aa^	0.07 ± 0.00^Bc^	0.03 ± 0.00^DEe^	0.05 ± 0.00 ^Cd^	0.19 ± 0.01^Aa^
p^1^	52.04 ± 0.08 ^H^	56.54 ± 0.17 ^F^	65.51 ± 0.18 ^C^	87.47 ± 0.22^A^	59.44 ± 0.28^E^	55.34 ± 0.34 ^G^	61.35 ± 0.20 ^D^	85.61 ± 0.33^B^

*E.s*. =* Ephedra sinica, G.u*. = *Glycyrrhiza uralensis, C.k. *=* Caragana korshinskii, A.m. *=* Allium mongolicum, T.v*. =* Thymus vulgaris, A.me*. =* Astragalus membranaceus, L.b. *= *Lespedeza bicolor*, DM = dry matter. The same superscript lowercase letters indicate *P* > 0.05; the different superscript lowercase letters indicate 0.01 < *P* < 0.05; the different superscript uppercase letters indicate *P* < 0.01. ^1^a is the rapid degrading components, b is the slow degrading components, c is the degradation rate of the slow degrading components, and p is the effective degradation rate.

### 3.6. Degradation of NDF of the herbs

**Figure 2.**
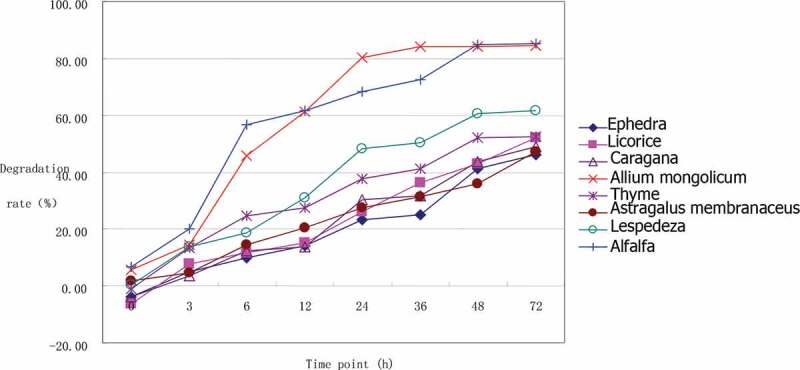
Ruminal neutral detergent fiber degradation of seven Chinese herbs

Digestibility of NDF is an important indicator of forage quality, which influences animal performance. The degradation rates of the NDF of the seven herbs at different time points calculated by the SAS nonlinear procedures are shown in Table 7 and Figure 2. After 72 h rumen degradation, the NDF degradation rate of *Ephedra* sinica was the lowest (46.33%), and that of Allium mongolicum was the highest (84.47%), with a difference of 38.47%. According to some reports [21], when the value of a forage is small, the degradation rate of NDF mainly depends on the b and c values, and an increase in c can improve the degradation rate. *A. mongolicum* and alfalfa have small a values while relatively high b and c values, so they have high effective degradation rates. Although *E. sinica, G. uralensis, C. korshinskii*, and *L. bicolor* have small a values, these also have small b and c values, so their effective degradation rates are also low.

**Table 7.** Ruminal neutral detergent fiber degradation of seven Chinese herbs (%)

NDF/DM	*E.s.*	*G.u.*	*C.k.*	*A.m.*	*T.v.*	*A.me.*	*L.b.*	Alfalfa
a^1^	0.06 ± 0.01^D^	−1.01 ± 0.14^Ec^	−1.55 ± 0.40 ^c^	1.62 ± 0.44^b^	3.43 ± 0.45^a^	3.50 ± 2.27 ^Ba^	1.98 ± 0.20^Cb^	5.45 ± 0.33^A^
b^1^	61.53 ± 2.85^Ca^	61.73 ± 1.94^a^	55.86 ± 1.48^D^	84.07 ± 0.71^A^	47.78 ± 0.64^Eb^	46.95 ± 1.35^b^	60.63 ± 0.55^a^	74.05 ± 0.65^B^
c^1^	0.02 ± 0.00 ^F^	0.03 ± 0.00^a^	0.03 ± 0.00^Ea^	0.10 ± 0.00^B^	0.06 ± 0.01^C^	0.03 ± 0.00^a^	0.06 ± 0.00^D^	0.13 ± 0.00^A^
p^1^	23.89 ± 0.29 ^G^	27.41 ± 0.37^Ea^	26.58 ± 0.20^Eb^	66.29 ± 0.30^A^	35.40 ± 0.20^D^	26.21 ± 0.53^Fb^	40.99 ± 0.43 ^C^	64.97 ± 0.78^B^

*E.s*. =* Ephedra sinica, G.u*. = *Glycyrrhiza uralensis, C.k. *=* Caragana korshinskii, A.m. *=* Allium mongolicum, T.v*. =* Thymus vulgaris, A.me*. =* Astragalus membranaceus, L.b. *= *Lespedeza bicolor*, DM = dry matter, NDF = neutral detergent fiber. The same superscript lowercase letters indicate *P* > 0.05; the different superscript lowercase letters indicate 0.01 < *P* < 0.05; the different superscript uppercase letters indicate *P* < 0.01. ^1^a is the rapid degrading components, b is the slow degrading components, c is the degradation rate of the slow degrading components, and p is the effective degradation rate.

### 3.7. Degradation of ADF of the seven herbs

**Figure 3.**
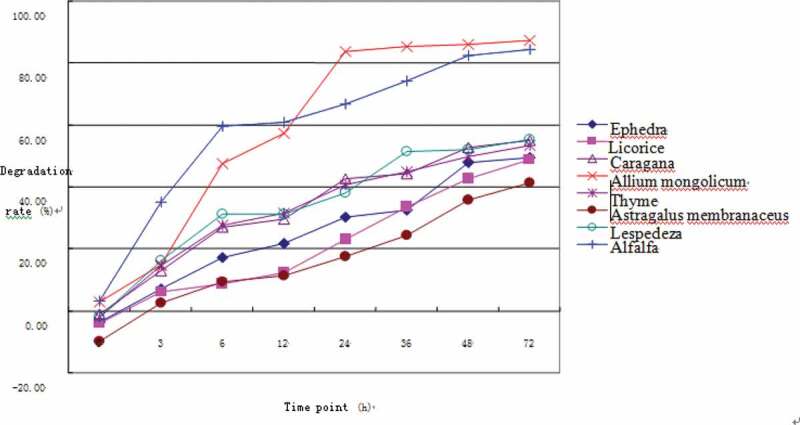
Ruminal acid detergent fiber degradation of seven Chinese herbs

Table 8 and Figure 3 show that the rumen degradation rates of ADF of the seven herbs gradually increased with rumen incubation time. *A. mongolicum* and alfalfa showed higher degradation of ADF than the other six herbs, with the effective ADF degradation rates of the latter <40%. The degradation rates of DM, NDF, and ADF can thus be used in evaluating the nutritional value of forages. The nutritional value of *A. mongolicum* was the highest among the seven herbs based on the degradation results. Ruminant digestion of fiber is of great significance, because of the high content of fiber in roughage, the fermentation product of fiber in the rumen is an important material and energy source for animals. The degradation rate of DM, NDF and ADF directly reflects the level of easily degradable substances. The comprehensive degradation rate of DM, NDF and ADF can basically make an objective evaluation of the nutritional value of a roughage.

**Table 8.** Ruminal acid detergent fiber degradation of seven Chinese herbs (%)

ADF/DM	*E.s.*	*G.u.*	*C.k.*	*A.m.*	*T.v.*	*A.me.*	*L.b.*	Alfalfa
a^1^	0.83 ± 0.08^Cb^	−1.12 ± 0.17^D^	2.05 ± 0.45 ^Ba^	0.26 ± 0.19^Cb^	0.59 ± 0.44^Cb^	−3.89 ± 0.37^E^	2.47 ± 0.44 ^Ba^	4.14 ± 0.28^A^
b^1^	50.54 ± 0.72^b^	63.83 ± 2.73 ^C^	50.19 ± 0.24^Eb^	87.64 ± 0.41^A^	48.36 ± 0.75^b^	53.31 ± 1.38 ^Da^	49.79 ± 0.84^b^	72.14 ± 0.45 ^B^
c^1^	0.04 ± 0.00^E^	0.02 ± 0.00^b^	0.08 ± 0.01^D^	0.10 ± 0.00 ^Ba^	0.10 ± 0.01^a^	0.02 ± 0.00^Fb^	0.09 ± 0.00^C^	0.19 ± 0.00^A^
p^1^	29.27 ± 0.15^D^	25.36 ± 0.40^E^	37.78 ± 0.30^Cc^	67.21 ± 0.49 ^Aa^	37.42 ± 0.69^Cc^	20.10 ± 0.48 ^F^	39.16 ± 0.23^B^	66.32 ± 0.43^Ab^

*E.s*. =* Ephedra sinica, G.u*. = *Glycyrrhiza uralensis, C.k. *=* Caragana korshinskii, A.m. *=* Allium mongolicum, T.v*. =* Thymus vulgaris, A.me*. =* Astragalus membranaceus, L.b. *= *Lespedeza bicolor*, DM = dry matter, ADF = acid detergent fiber. The same superscript lowercase letters indicate *P* > 0.05; the different superscript lowercase letters indicate 0.01 < *P* < 0.05; the different superscript uppercase letters indicate *P* < 0.01. ^1^a is the rapid degrading components, b is the slow degrading components, c is the degradation rate of the slow degrading components, and p is the effective degradation rate.

### 3.8. Degradation of CP of the Seven herbs

**Figure 4.**
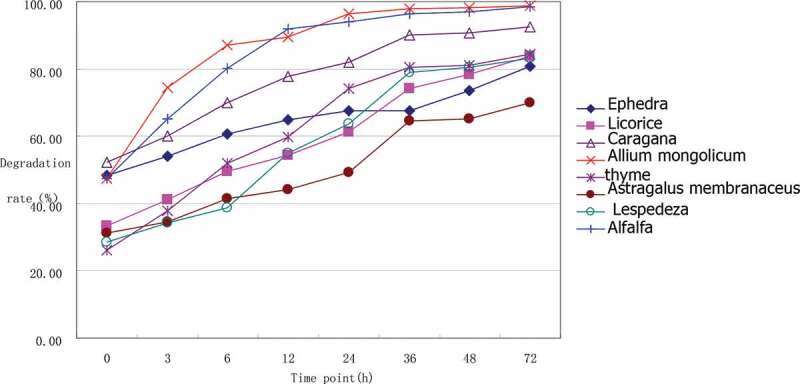
Ruminal crude protein degradation of seven Chinese herbs

The ruminal CP degradation results of the seven herbs as presented in Table 9 and Figure 4 indicate that the degradation of CP in the rumen was mainly completed within 36 h after feeding. Dietary proteins that have escaped ruminal degradation, together with proteins synthesized by rumen microorganisms, represent the primary source of amino acids that are absorbed by the small intestine [22].CP digestibility depends on the stage of forage maturity [23], forage species [24], and preservation methods [25]. The observed differences in CP degradation rates of the seven herbs and alfalfa may be attributable to differences among species. The lower lignin content of *A. mongolicum* and alfalfa may also contribute to their high degradation rate.

**Table 9.** Ruminal crude protein degradation of seven Chinese herbs (%)

CP/DM	*E.s.*	*G.u.*	*C.k.*	*A.m.*	*T.v.*	*A.me.*	*L.b.*	Alfalfa
a^1^	51.85 ± 0.36^Aa^	35.83 ± 0.12^Cc^	52.86 ± 0.32^a^	48.05 ± 1.71^Bb^	26.94 ± 0.61^e^	31.56 ± 1.22^Dd^	27.03 ± 0.42^Ee^	46.71 ± 0.98^b^
b^1^	28.81 ± 1.44 ^G^	51.82 ± 0.67^Ca^	38.68 ± 0.12 ^F^	48.99 ± 1.80 ^Db^	56.43 ± 0.69^B^	45.11 ± 1.01^E^	59.81 ± 0.26^A^	50.32 ± 0.78^CDab^
c^1^	0.03 ± 0.00^Dec^	0.04 ± 0.00 ^c^	0.08 ± 0.00^Ca^	0.24 ± 0.01^A^	0.08 ± 0.00^a^	0.03 ± 0.00^Ec^	0.05 ± 0.00 ^Db^	0.17 ± 0.01^B^
p^1^	66.82 ± 0.09^E^	63.41 ± 0.14 ^F^	80.68 ± 0.11^C^	91.52 ± 0.14^A^	67.52 ± 0.22^D^	53.12 ± 0.38 ^H^	62.80 ± 0.16 ^G^	89.38 ± 0.23^B^

*E.s*. =* Ephedra sinica, G.u*. = *Glycyrrhiza uralensis, C.k. *=* Caragana korshinskii, A.m. *=* Allium mongolicum, T.v*. =* Thymus vulgaris, A.me*. =* Astragalus membranaceus, L.b. *= *Lespedeza bicolor*, DM = dry matter, CP = crude protein. The same superscript lowercase letters indicate *P* > 0.05; the different superscript lowercase letters indicate 0.01 < *P* < 0.05; the different superscript uppercase letters indicate *P* < 0.01.

^1^a is the rapid degrading components, b is the slow degrading components, c is the degradation rate of the slow degrading components, and p is the effective degradation rate.

## 4. Conclusions

The content of amino acids, fatty acids, and mineral elements, as well as the dynamic degradation rates of DM, NDF, ADF, and CP of seven Chinese herbs in the rumen of Tan sheep showed that all the seven herbs meet the nutritional needs of ruminants based on the standard forage alfalfa. However, *Ephedra, Glycyrrhiza, Caragana, Allium, Astragalus*, and *Lespedeza* have higher nutritive value than *Thymus*. According to the ruminal degradation rates of DM, NDF, ADF, and CP, the nutritive values of *Caragana, Allium*, and *Lespedeza* are higher than those of *Ephedra, Glycyrrhiza, Astragalus*, and *Thymus*. The overall nutritive value of *Allium* is the highest among the seven herbs and therefore it is a recommended forage to better meet the nutritional needs of Tan sheep.

## Article highlights

1. Impact of seven herbs of traditional Chinese medicine in Tan sheep digestion was studied.
2. Digestion of the rumen of Tan sheep was examined using the nylon bag method.
3. All seven herbs were met the nutritional needs of ruminants based on the standard forage alfalfa.
4. Overall nutritive value of Allium is the highest among the seven herbs.

## References

[1] Li H, Ge CC, Feng F, et al. Fatty acids in longissimus dorsi from fattening Tan sheep, Small Fat-tail sheep and Tan Han hybrid sheep[J]. Food Res Develop. 2019;40(3):165–169.

[2] Hu YX. Development status and Countermeasures of Tan sheep industry in Yanchi County [J]. Modern Agric Sci Technol. 2017;30(12):261–263.

[3] Liu YN, Guo CH, Zhang ZF. Research progress in the application of Chinese herbal feed additives in the diet of pregnant and lactating ewes [J]. Chin J Herbivorous Animal Sci. 2018;38(1):57–59.

[4] Yang ZH. Multiple measures and pilot projects to accelerate the development of grass and animal husbandry[J]. Pratacultural Sci. 2015;32(8):1201–1205.

[5] Huang WY. Quality analysis of Tibetan sheep grazing on alpine meadow grassland in Qinghai Pastoral Area [J]. Meat Res. 2015;76(5):1212–1215.

[6] Zhang J. Nutritional value evaluation of grassland forage in different months in Naqu City, Tibet and Study on nutritional supplement of cashmere goats [D]. Ya’an: Sichuan Agricultural University; 2005.

[7] Han YW. Feed and feeding [M]. Beijing: China Agricultural Press; 1998. p. 24–39.

[8] Mehrez AZ, Orskov ER. A study of artificial fiber bag technique for determining the digestibility of feeds in the rumen[J]. J Agric Sci. 1977;88(6):645–650.

[9] Ji ZH, Wang SY. Animal husbandry and Veterinary Bureau of the Ministry of agriculture (national feed work office).Compilation of feed industry standards[M]. Vols. 70-72. Beijing: China Standard Press; 2002. p. 79–80.

[10] Van Soest PJ, Robertson JB, Lewis BA. Methods for dietary fiber, neutral detergent fiber, and nonstarch polysaccharides in relation to animal nutrition[J]. J Dairy Sci. 1991;74(10):3583–3597. 1660498 10.3168/jds.S0022-0302(91)78551-2

[11] Chinese Standard GB/T 18246–2000. Cereal crop and forage crop in general: Determination of amino acids in feeds. Beijing, China: Standards Press of China;2008.

[12] Chinese Standard GB/T22223. Determination of total fat, saturated fat, and unsaturated fat in foods: Hydrolytic extraction-gas chromatography. Beijing, China: Standards Press of China; 2008.

[13] Chinese Standard GB/T18932.12. Method for the determination of potassium, sodium, calcium, magnesium, zinc, iron, copper, manganese, chromium, lead, cadmium contents in honey: atomic absorption spectrometry. Beijing,China: Standards Press of China; 2002.

[14] Chinese Standard GB/T13883. Determination of selenium in feeds. Beijing,China: Standards Press of China; 2008.

[15] Chinese Standard GB13078. Hygienical standard for feeds. Beijing,China: Standards Press of China; 2001.

[16] Zhai SW. Effects of dietary protein levels on urea nitrogen concentration and nitrogen utilization efficiency of dairy cows [J]. Dairy Sci Technol. 2008;33(6):266–268.

[17] FAO/WHO. Energy and protein requirement. Gneve: WHO; 1973. p. 631.

[18] Wood JD, Enser. Factors influencing fatty acids in meat and the role of antioxidants in improving meat quality[J]. Br J Nutr. 1997;78(1):49–60. 10.1079/bjn19970134 9292774

[19] Sayanova OV, Napier JA. Eicosapentaenoic acid: biosynthetic routes and the potential for synthesis in transgenic plants[J]. Phytochemistry. 2004;65:147–158. 14732274 10.1016/j.phytochem.2003.10.017

[20] Nuyen LQ, Nuijensm CGA, Everts H, et al. Mathematical relationships between the intake of n6 and n3 polyunsaturated fatty acids and their contents in adipose tissue of growing pigs[J]. Meat Sci. 2003;65(4):1399–2140. 22063784 10.1016/S0309-1740(03)00062-7

[21] Hoffman PC, Sievert SJ, Shaver RD, et al. In situ dry matter, protein, and fiber degradation of perennial forages[J]. Dairy Sci. 1993;48(76):2632–2643. 10.3168/jds.S0022-0302(93)77599-2 8227665

[22] Taghizadeh A, Danesh MM, Valizadeh RF, et al. Digestion of feed amino acids in the rumen and intestine of steers measured using a mobile nylon bag technique[J]. Dairy Sci. 2005;88(88):1807–1814. 10.3168/jds.S0022-0302(05)72855-1 15829674

[23] Cherney DJ, Cherney JH. Grass forage quality and digestion kinetics as influenced by nitrogen fertilization and maturity[J]. Animal Res. 1997;2(11):105–120.

[24] Janicki FJ, Stallings CC. Degradation of crude protein in forages determined by in vitro and in situ procedures[J]. Dairy Sci. 1988;71(71):2440–2448.

[25] Tamminga S, Ketelaar R. Degradation of nitrogenous compounds in conserved forages in the rumen of dairy cows[J]. Grass Forage Sci. 1991;22(46):427–435.

